# Association of Circulating IGFBP1 Level with the Severity of Coronary Artery Lesions in Patients with Unstable Angina

**DOI:** 10.1155/2017/1917291

**Published:** 2017-02-20

**Authors:** Wei Zheng, Yayu Lai, Peng Jin, Wenzhu Gu, Qi Zhou, Xiaojing Wu

**Affiliations:** ^1^Cardiovascular Department, Xinqiao Hospital, Third Military Medical University, Chongqing, China; ^2^Cardiovascular Department, The Second Affiliated Hospital of Chongqing Medical University, Chongqing, China

## Abstract

*Aims.* Local IGFBP1 level was reported to affect the development of coronary artery plaque. This study investigated the association of circulating IGFBP1 level with the severity of coronary artery lesions in patients with unstable angina.* Materials and Methods.* In 112 consecutive patients with clinically diagnosed unstable angina, admitted from July 2014 to July 2015, we studied the correlations of circulating IGFBP1 and the severity of coronary artery disease (CAD).* Results.* All patients underwent scheduled coronary angiography, and 67 cases were diagnosed with critical and 45 with noncritical CAD. Of the 67 critical CAD patients, 41 (61.19%) presented with multivessel and 26 (38.81%) with single-vessel lesions. IGFBP1 levels were higher in patients with multivessel than those with single-vessel lesions. Moreover, the IGFBP1 level was positively correlated with the GRACE score. Among clinical variables, the IGFBP1 level was correlated with HDL-C. IGFBP1 alone (cutoff 20.86 ng/ml) demonstrated a sensitivity of 0.448 and specificity of 0.933 in predicting CAD. Combination of IGFBP1 and HDL-C had a sensitivity of 0.821 and specificity of 0.800 in predicting CAD.* Conclusions.* Circulating IGFBP1 level positively correlated with the severity of CAD. IGFBP1, when combined with HDL-C, might be useful in screening for high risk CAD patients.

## 1. Introduction

Chest pain is a common symptom in an emergency department. Several disorders, including coronary artery disease (CAD), gastroesophageal reflux disease, diseases of pleura, and pulmonary vessels, can cause chest pain [1]. How to screen high risk chest pain patients due to acute coronary syndrome (ACS) is the key to effectively manage life-threatening CAD. Typical electrocardiogram (ECG) and cardiac necrosis biomarkers are helpful in the detection of myocardial infarction (MI). However, their utility in detecting patients with unstable angina but without typical ECG or enzyme changes is very limited. The development of coronary plaque is a major precipitating factor in the triggering of ACS [2]. The traditional biomarkers that reflect myocardial necrosis represent the severe and late stages of ACS and may not serve as indicators for all subtype patients with ACS in a clinical practice. Search for novel biomarkers that are directly linked to the severity of coronary artery lesions is warranted [3].

Insulin-like growth factor (IGF) binding protein 1 (IGFBP1) is a ~30 kDa secretory protein produced mainly in the liver and kidney and with lesser expression in other tissues [4]. It belongs to the IGFBPs family and modulates IGF bioactivity through its high-affinity binding. Earlier studies showed that circulating level of IGFBP1 was associated with insulin resistance and diabetes [5, 6]. However, more recently, studies reported that IGFBP1 affects the prognosis and mortality of cardiovascular diseases in patients without diabetes and glucose intolerance [7–9], which might be due to an independent direct regulation of vascular endothelial cells (EC) and smooth muscle cells (SMC) [4]. Studies by Wang et al. [10] showed that IGFBP1 stimulated SMC proliferation through extracellular signal-regulated kinase-1/2 (ERK1/2) activation. Rajwani et al. [11] found that IGFBP1 increased endothelial nitric oxide synthase activity in EC via phosphatidylinositol 3-kinase signaling. Both studies reported that IGFBP1 exerts IGF-independent effects at the cellular level. In particular, IGFBP1 is expressed at very low levels in normal vessel wall but has been found increased in atherosclerotic plaques [12]. Wang et al. [10] reported that increased levels of IGFBP1 were detected in human carotid plaques and might play a role in fibroproliferative processes and contribute to plaque instability. However, very little is known about the correlation of circulating IGFBP1 and the severity of coronary artery lesions. We therefore hypothesized that the circulating IGFBP1 level might be associated with the extent of the atherosclerotic lesions and could be used as a biomarker reflecting the severity of coronary artery lesions in patients with unstable angina.

## 2. Methods

### 2.1. Patients and Study Design

We consecutively collected data from patients who were clinically diagnosed with unstable angina at Xinqiao Hospital between July 2014 and July 2015. The diagnosis of unstable angina referred to the recent guidelines [13, 14]. Patients with cardiac valve diseases, congenital heart diseases, aortic aneurysm, connective tissue diseases, infections, chronic kidney diseases, and cancer were excluded. Patients with previous percutaneous coronary intervention or coronary artery bypass grafting were also excluded. Because IGFBP1 levels were variable among individuals with a varying glucose tolerance, we excluded the patients with diabetes and impaired glucose tolerance. Finally 112 patients with unstable angina but without typical changes of ECG and troponin were enrolled. The laboratory and demographic data and disease history were collected at the time of admission. Fasting peripheral venous blood was drawn from all enrolled subjects for the assay of IGFBP1 and routine analysis of blood. The study protocol conforms to the ethical guidelines of the 1975 Declaration of Helsinki and has been approved by the institutional ethical committee of Xinqiao Hospital, the Third Military Medical University. Informed consent was obtained from all enrolled subjects.

### 2.2. The Severity Assessment of CAD

All patients underwent scheduled coronary angiography (CAG) according to standard techniques, and the severity of coronary lesions was assessed by at least 2 experienced interventional cardiologists. Critical CAD was defined as a segment of ≥50% stenosis in any major epicardial artery or any important branch of a major epicardial coronary artery. The severity of CAD was assessed by the number of diseased vessels and the value of the Global Registry of Acute Coronary Events (GRACE) risk score.

Single-vessel disease was defined as a ≥50% stenosis in only one major epicardial artery or an important branch of a major epicardial coronary artery. Multivessel coronary disease was defined as the presence of a >50% stenosis in two or more major epicardial arteries or left main coronary artery. For each patient, the GRACE score was calculated by using eight specific variables collected at admission as previously reported (http://www.gracescore.org/website/webversion.aspx) [15, 16]. Patients were categorized as low risk (GRACE score ≤ 108) versus intermediate and high risk (GRACE score > 108).

### 2.3. ELISA Measurement of IGFBP1

The serum levels of IGFBP1 were determined using a commercially available human IGFBP1 ELISA kit from RayBiotech (USA). Briefly, approximately 3 ml of blood was collected in a vacuum tube after an overnight fast. Blood was centrifuged to obtain serum. Serum samples were stored at −70°C until the concentration of IGFBP-1 was measured. Assays were performed according to the manufacturer's instructions. Samples were measured in duplicate.

### 2.4. Biochemical Parameters

Routine biochemical parameters including levels of lipids, blood glucose, liver, and renal function were assayed using a Beckman Coulter AU5800 system (USA). The level of B-type natriuretic peptide (BNP) was assayed by a ReLIA Analyzer SSJ-2 using the reagents provided by the manufacturer (ReLIA Biotechnologies, USA). Blood pressure (BP) and heart rate were measured with an Omron HEM-6200 monitor (Japan).

### 2.5. Statistical Analysis

Statistical analysis was performed using SPSS software (version 20.0, IBM Corp., America). Continuous variables are summarized as the mean ± SD or medians and interquartile ranges, and all categorical variables are expressed as proportions. Statistical significance of the differences of the biomarker levels between the groups was determined using a Kruskal-Wallis nonparametric test. Correlations between IGFBP1 levels and severity of coronary artery lesions were assessed by calculating Spearman correlation coefficients. Predictive factors were examined by univariate and multivariate analyses. The prognostic accuracies of serum biomarker levels were assessed by receiver operating characteristic (ROC) curve analysis. Forward stepwise binary logistic regression analyses were performed to determine the optimal combination of biomarkers for predicting CAD. *P* < 0.05 was considered statistically significant.

## 3. Results

### 3.1. Patient Characteristics and Circulating Level of IGFBP1

**Figure 1:**
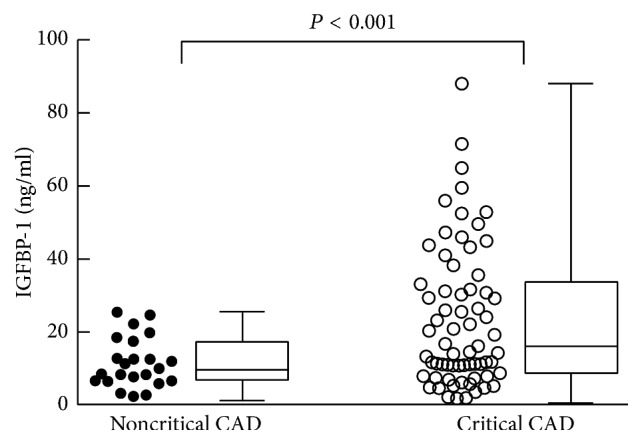
Comparison of circulating IGFBP-1 level between the critical CAD and noncritical CAD group. CAD = coronary artery disease; IGFBP1 = insulin-like growth factor binding protein 1.

In the 112 enrolled patients with unstable angina, 67 patients were diagnosed by CAG with critical CAD and 45 with noncritical CAD. The age distribution was comparable between the two groups. Consistent with other cardiovascular studies, the ratio of male gender and cigarette smoking was higher in the critical CAD group than those in the noncritical control group (*P* < 0.05). Body mass index (BMI) in the critical CAD group was higher than that in the noncritical CAD group. The blood pressure, levels of low density lipoprotein cholesterol (LDL-C), and total cholesterol (TC) were comparable between the critical CAD group and the noncritical CAD group. The levels of high density lipoprotein cholesterol (HDL-C) were lower while BNP levels were higher in the critical CAD group than those in the noncritical control group. The average IGFBP1 level was higher in the critical CAD group (16.12 ng/ml, IQR 8.74–33.20 ng/ml) than that in the noncritical control group (10.02 ng/ml, IQR 5.82–12.98 ng/ml) (*P* < 0.001) (Table 1, Figure 1).

### 3.2. Correlations of IGFBP1 with Other CAD Risk Factors

Because IGFBP1 was differentially expressed between the critical CAD group and noncritical control group, we further analyzed its correlation with other CAD risk factors. There was no correlation of IGFBP1 level with gender, cigarette smoking, TC, TG, and LDL-C. However, the IGFBP1 level was positively correlated with age (*r* = 0.505, *P* < 0.001), HDL-C (*r* = 0.212, *P* < 0.05), and BNP (*r* = 0.343, *P* < 0.001) (Table 2).

### 3.3. Correlation of IGFBP1 Level and Coronary Artery Lesions

**Figure 2:**
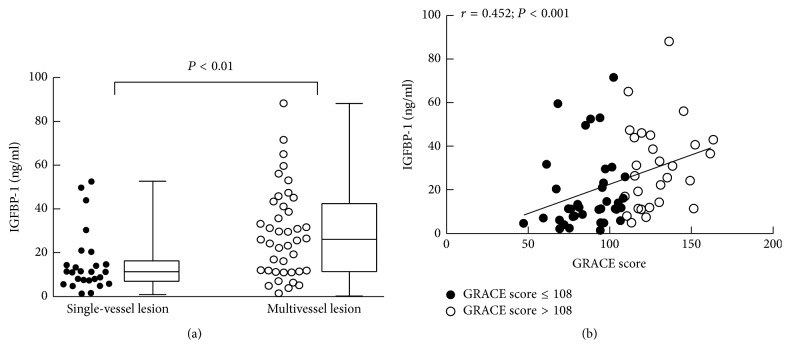
Correlations of circulating IGFBP-1 level with the severity of coronary artery lesions. (a) Circulating IGFBP1 level was higher in patients with multivessel lesions than those in single-vessel lesion; (b) circulating IGFBP1 level was positively correlated with GRACE score. GRACE = the global registry of acute coronary events; IGFBP1 = insulin-like growth factor binding protein 1.

Of the 67 patients with critical CAD, 41 patients (61.19%) were found to have multivessel lesions and 26 (38.81%) have single-vessel lesion. The average level of IGFBP1 was higher in patients with multivessel lesions (26.08 ng/ml, IQR, 11.63–42.26 ng/ml) than those with single-vessel lesion (11.26 ng/ml, IQR, 7.01–16.13 ng/ml, *P* < 0.01). In addition, the level of IGFBP1 was positively correlated with the GRACE score (*r* = 0.452, *P* < 0.001) (Figure 2).

### 3.4. IGFBP1 Was an Independent Predicting Factor for CAD

Univariate and multivariate analyses were carried out to identify the factors related to CAD. We used the univariate analysis to select the significant risk factors of CAD, and the results showed that male gender (OR 5.331, 95% CI 2.344–12.126, *P* < 0.001), BMI (OR 1.190, 95% CI 1.025–1.381, *P* < 0.05), cigarette smoking (OR 3.194, 95% CI 1.411–7.228, *P* < 0.01), HDL-C (OR 0.052, 95% CI 0.010–0.277, *P* < 0.01), BNP (OR 1.015, 95% CI 1.004–1.026, *P* < 0.01), and IGFBP1 (OR 1.091, 95% CI 1.041–1.144, *P* < 0.001) were significantly related to CAD. Risk factors selected from the univariate analysis were used as variables of the forward stepwise multivariate logistic regression mode. Cigarette smoking, HDL-C, and IGFBP1 were further entered into the multivariate logistic regression analysis. IGFBP1 (OR 1.170, 95% CI 1.080–1.268, *P* < 0.001) was identified as an independent predictive factor for CAD (Table 3).

### 3.5. ROC Analysis

The prognostic accuracies of each biochemistry marker determined by the ROC curve analysis were shown in Table 4. IGFBP1 alone (cutoff 20.86 ng/ml) demonstrated a sensitivity of 0.448 and specificity of 0.933 with an area under the curve (AUC) of 0.719 in predicting CAD. Forward stepwise binary logistic regression analysis showed that combination of IGFBP1 and HDL-C demonstrated a better predictive value, with an AUC of 0.865 and Youden index of 0.621, than each individual biomarker. The logistic formula *P* = 1/(1 + exp⁡(−*Z*)) was used to calculate the probability of IGFBP1 and HDL-C in predicting CAD. *Z* = *β*_0_ + *β*_1_*X*_1_ + *β*_2_*X*_2_ + ⋯+*β*_*m*_*X*_*m*_. *β*_0_ is a constant, and *β*_1_, *β*_2_,…, *β*_*m*_ are estimated regression coefficients of the risk factors. So the predicted probability was calculated as follows:

(1) $$\begin{array}{c} P=\frac{1}{1+{exp}^{-\left(4.198+0.145\times \left[IGFBP\text{-}1\right]-5.251\times \left[HDL\right]\right)}}. \end{array}$$

**Figure 3:**
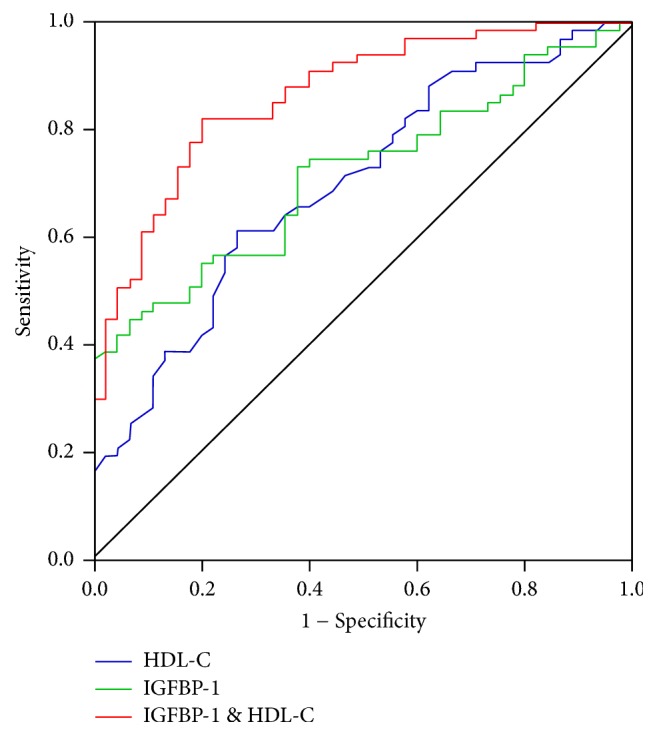
Receiver operating characteristic (ROC) curve analysis of IGFBP-1, HDL-C, or combination of both in predicting CAD. ROC = receiver operating characteristic; HDL-C = high density lipoprotein cholesterol, CAD = coronary artery disease; IGFBP1 = insulin-like growth factor binding protein 1.

Combination of IGFBP1 and HDL-C (cutoff 0.511) had a sensitivity of 0.821 and specificity of 0.800 in predicting CAD (Figure 3, Table 4).

## 4. Discussion

Chest pain suggestive of ACS is a common cause of presentation with poor prognosis in the emergency department [1, 2]. Diagnosis based on 18-lead ECG and traditional cardiac necrosis biomarkers including troponin recognizes less than half of the patients with MI. However, their ability to predict the severity of coronary lesions in patients with unstable angina is limited. Among the patients with chest pain, the diagnosis of CAD was ruled out in up to one-third of patients [17, 18]. Although invasive CAG is currently the gold standard for the diagnosis of CAD, the discovery of circulating biomarkers both affecting the development of atherosclerotic plaque and reflecting the severity of coronary artery lesions may provide a simple and fast measure recognizing high risk patients.

The development of atherosclerosis involves complex processes, including inflammation, thrombosis, and abnormal lipids. The rupture or erosion of coronary plaque triggers the onset of ACS [2]. Among the mechanisms affecting a development of plaque, locally expressed IGFBP1 has been reported to be increased in atherosclerotic plaques and contribute to the plaque stability. Since IGFBP1 is a secretory protein, its corresponding changes in circulation in the case of ACS are not well studied. In this study, we studied 112 patients diagnosed with unstable angina. Among them, 67 patients were diagnosed by CAG with critical CAD and 45 with noncritical CAD. The average IGFBP1 level was higher in the critical CAD group than that in the noncritical CAD group. Further, of the 67 patients with critical CAD, 41 patients (61.19%) were found with multivessel and 26 (38.81%) with single-vessel lesion. The average level of IGFBP1 was higher in the patients with multivessel lesions than that in those with single-vessel lesion. Consistent with previous studies [7–9], we found that IGFBP1 was positively correlated with age. Nevertheless, in age matched patients, the level of IGFBP1 was higher in patients with multivessel lesions than those with single-vessel lesion. Although low values of IGFBP1 may not exclude CAD, it is very likely that high levels of IGFBP1 might help recognize high risk CAD patients. Our study showed that increased circulating levels of IGFBP1 correlated with the severity of coronary artery lesions, raising the possibility that IGFBP1 may serve as a potential biomarker for the severity of CAD.

GRACE score is a predictive logistical model which uses 8 prognostic variables to estimate the risk of death or the combined risk of death and MI in individual patient. Besides the original function for risk stratification, recent studies showed that the GRACE score has significant value for assessing the severity and extent of coronary artery stenosis in patients with ACS [19]. Patients with high GRACE score have more severe CAD. In our study, the level of IGFBP1 was positively correlated with the GRACE score, suggesting a link between IGFBP1 level and the severity of CAD.

To further evaluate the role of IGFBP1 in predicting CAD, we analyzed its correlation with the traditional coronary risk factors. We found that there was no correlations of IGFBP1 level with gender, cigarette smoking, TC, TG, and LDL-C. However, the IGFBP1 level was positively correlated with HDL-C and BNP, respectively. In the multivariate logistic regression analysis, with smoking and low HDL-C level, IGFBP1 was identified as an independent predictive factor for CAD.

HDL-C is a biomarker for lipids and high levels of HDL-C are associated with reduced risk of atherosclerosis [20, 21]. Although the clinical outcome involving HDL-raising drugs is disappointing [22, 23], the risk predicting value of HDL-C for CAD has been well recognized. In this study, we found that IGFBP1 alone demonstrated a low sensitivity and a high specificity in predicting CAD. However, combination of HDL-C can improve the predictive accuracy of IGFBP1. Due to the complexity of the CAD disease process, the potential for biomarker combinations is currently of considerable interest in early diagnosis and prognosis. Our study showed that combination of IGFBP1 and HDL-C demonstrated a better predictive value than each of the individual biomarker and might serve as an optimal panel for screening high risk CAD patients.

So far the role of IGFBP1 in cardiovascular prognosis is controversial. Studies from Rajwani et al. [11] and Borai et al. [24] showed that increased IGFBP1 levels protected against atherosclerosis and low levels of IGFBP1 could be a marker of coronary risk. Study from Wang et al. [10] reported that increased levels of IGFBP1 were detected in human carotid plaques and might play a role in fibroproliferative processes and contribute to plaque instability. In contrast, other evidence showed that increased levels of IGFBP1 were usually associated with the release of inflammation factors, which correlated with poor prognosis and high mortality rate [6, 7]. We found that high circulating level of IGFBP1 was associated with the severity of coronary artery lesions. However, whether IGFBP1 is a harmful or endogenous protective factor is still debatable.

There are several limitations to this study. Firstly, the major limitation of this study was the relatively low number of enrolled subjects. An expanded random sample across the gender and age range may be more representative. Long term follow-up of the changing pattern of IGFBP1 as well as its criteria in the assessment of coronary artery lesions among individual subjects with different age warrant extensive studies. Secondly, the severity of CAD was elevated by the number of diseased vessels and the value of GRACE score. Additional quantitative tools including SYNTAX score might provide more information on the complexity of coronary artery lesions and should be considered in future studies. Finally, as an ideal diagnostic tool the aim is to screen for the disease even when it is in the less severe form. Our data indicated a link between circulating IGFBP1 level and the severity of CAD. While high levels of IGFBP1 might help recognize high risk CAD patients, low values of IGFBP1 may not definitively exclude CAD. Further investigations are warranted to determine if these preliminary results can be expanded to detect high-risk compared to lower risk in CAD patients.

In conclusion, as a regulator in the development of coronary plaques, circulating IGFBP1 showed a great promise in predicting the severity of coronary artery lesions in patients with unstable angina. With cigarette smoking and HDL-C level, IGFBP1 was identified as an independent predictor for CAD. IGFBP1, especially when combined with HDL-C, may serve as a potential biomarker for screening high risk CAD patients.

## Figures and Tables

**Table 1:** Basic characteristics of the enrolled subjects.

	Noncritical CAD (*n* = 45)	Critical CAD (*n* = 67)	*P* value
*Age, year*	60.11 ± 9.91	62.16 ± 11.08	0.318
*Gender, male, n (%)*	16 (35.60)	50 (74.60)	<0.001
*BMI, kg/m* ^*2*^	23.03 ± 2.23	24.28 ± 3.03	0.019
*Cigarette smoking, n (%)*	12 (26.70)	36 (53.70)	0.005
*Hypertension, n (%)*	21 (46.70)	33 (49.30)	0.788
SBP, mmHg	132.98 ± 15.91	132.28 ± 19.72	0.844
DBP, mmHg	78.40 ± 10.74	75.37 ± 11.65	0.167
*Hyperlipidemia*	16 (35.60)	19 (28.40)	0.420
TC, mmol/L	4.39 ± 0.98	4.05 ± 0.96	0.070
TG, mmol/L	1.22 (0.87, 1.64)	1.27 (1.00, 1.76)	0.251
ApoB, g/L	0.78 ± 0.22	0.76 ± 0.20	0.781
HDL-C, mmol/L	1.22 ± 0.28	1.02 ± 0.25	<0.001
LDL-C, mmol/L	2.66 ± 0.76	2.48 ± 0.68	0.183
*Medication*			
Antiplatelets, *n* (%)	31 (68.90)	64 (95.50)	<0.001
ACEIs or ARBs, *n* (%)	26 (57.80)	49 (73.10)	0.090
*β*-blockers, *n* (%)	31 (68.90)	45 (67.20)	0.848
CCB, *n* (%)	14 (31.10)	8 (11.90)	0.012
Statins, *n* (%)	31 (68.90)	64 (95.50)	<0.001
Diuretics, *n* (%)	6 (13.30)	8 (11.90)	0.827
*Glucose, mmol/L*	4.75 ± 0.64	4.69 ± 0.58	0.601
*BNP, pg/ml*	16.10 (6.55, 25.50)	46.60 (15.40; 85.00)	<0.001
*IGFBP-1, ng/ml*	10.02 (5.82; 12.98)	16.12 (8.74; 33.20)	<0.001

CAD: coronary artery disease; BMI: body mass index; SBP: systolic blood pressure; DBP: diastolic blood pressure; TC: total cholesterol; TG: total triglyceride; ApoB: apolipoprotein B; HDL-C: high density lipoprotein cholesterol; LDL-C: low density lipoprotein cholesterol; ACEIs: angiotensin-converting enzyme inhibitors; ARBs: angiotensin receptor blockers; CCB: calcium channel blockers; BNP: brain natriuretic peptide; IGFBP-1: insulin-like growth factor binding protein-1.

**Table 2:** Correlations between IGFBP-1 and other CAD risk factors.

	Correlation coefficients	*P* value
Age	0.505	<0.001
Gender (male)	0.131	0.168
Cigarette smoking	0.000	1.000
BMI	−0.201	0.033
TC	0.005	0.958
TG	−0.141	0.138
ApoB	−0.024	0.798
HDL-C	0.212	0.025
LDL-C	−0.003	0.978
BNP	0.343	<0.001

CAD: coronary artery disease; BMI: body mass index; TC: total cholesterol; TG: total triglyceride; ApoB: apolipoprotein B; HDL-C: high density lipoprotein cholesterol; LDL-C: low density lipoprotein cholesterol; BNP: brain natriuretic peptide; IGFBP-1: insulin-like growth factor binding protein-1.

**Table 3:** Logistic regression analysis.

Variables	Univariate logistic regression			Multivariate stepwise logistic regression		
	Estimate	OR (95% CI)	*P* value	Estimate	OR (95% CI)	*P* value
*Gender, male*	1.674	5.331 (2.344, 12.126)	<0.001	—	—	0.630
*BMI, kg/m* ^*2*^	0.174	1.190 (1.025, 1.381)	0.022	—	—	0.052
*Cigarette smoking*	1.161	3.194 (1.411, 7.228)	0.005	1.203	3.331 (1.171, 9.477)	0.024
*Hypertension*	0.104	1.109 (0.521, 2.364)	0.788	—	—	—
SBP, mmHg	−0.002	0.988 (0.977, 1.019)	0.998	—	—	—
DBP, mmHg	−0.024	0.976 (0.943, 1.010)	0.168	—	—	—
*Hyperlipidemia*	−0.332	0.717 (0.319, 1.611)	0.421	—	—	—
TC, mmol/L	−0.368	0.692 (0.462, 1.037)	0.074	—	—	—
TG, mmol/L	0.292	1.339 (0.739, 2.426)	0.336	—	—	—
ApoB, g/L	−0.261	0.770 (0.125, 4.749)	0.779	—	—	—
HDL-C, mmol/L	−2.956	0.052 (0.010, 0.277)	0.001	−5.043	0.006 (0.001, 0.080)	<0.001
LDL-C, mmol/L	−0.367	0.693 (0.403, 1.192)	0.185	—	—	—
*BNP, pg/ml*	0.015	1.015 (1.004, 1.026)	0.008	—	—	0.144
*IGFBP-1, ng/ml*	0.088	1.091 (1.041, 1.144)	<0.001	0.157	1.170 (1.080, 1.268)	<0.001

OR: odds ratio; BMI: body mass index; SBP: systolic blood pressure; DBP: diastolic blood pressure; TC: total cholesterol; TG: total triglyceride; ApoB: apolipoprotein B; HDL-C: high density lipoprotein cholesterol; LDL-C: low density lipoprotein cholesterol; BNP: brain natriuretic peptide; IGFBP-1: insulin-like growth factor binding protein-1.

**Table 4:** Summary of ROC Curves.

Parameter	AUC (95% CI)	SE	*P* value	Cutoff	Sensitivity	Specificity	Youden index
IGFBP-1	0.719 (0.626, 0.811)	0.047	<0.001	>20.861	0.448	0.933	0.381
HDL-C	0.699 (0.601, 0.796)	0.050	<0.001	<1.065	0.612	0.733	0.345
IGFBP-1 & HDL-C	0.865 (0.799, 0.931)	0.034	<0.001	>0.511	0.821	0.800	0.621

ROC: receiver operating characteristic; AUC: area under ROC curve, SE: standard error, CI: confidence interval; IGFBP-1: insulin-like growth factor binding protein-1; HDL-C: high density lipoprotein cholesterol.
